# Comprehensive Rehabilitation of Charcot Foot With Equinovarus Deformity: A Case Report

**DOI:** 10.7759/cureus.48431

**Published:** 2023-11-07

**Authors:** Neha M Chitlange, Sakshi Padmawar, Pratik Phansopkar

**Affiliations:** 1 Department of Musculoskeletal Physiotherapy, Ravi Nair Physiotherapy College, Datta Meghe Institute of Higher Education & Research (DMIHER), Wardha, IND

**Keywords:** achilles tendon lengthening, physical therapy, post-traumatic talipes equinovarus, equinovarus deformity, charcot neuroarthropathy

## Abstract

Charcot neuroarthropathy is the destruction of the bones and joints caused by underlying neuropathy, trauma, and disturbances in bone metabolism. Modern health care and surgical options now include limb salvage. An acquired or congenital foot deformity is the equinovarus deformity, also known as clubfoot or talipes equinovarus. The foot is fixed in plantarflexion (equinus), deviates toward its center (varus), and is rotated upward so that it rests on its outside (supination) in this condition. In another way, the foot turns axially outward while pointing downward and inward. Charcot neuroarthropathy generally occurs due to diabetes, but in this case, it occurs due to trauma and leads to a traumatic congenital talipes equinovarus deformity. A 38-year-old male patient complained of right foot pain and an inability to walk. Two years ago, he was involved in an accident that left him with a right leg injury. He was eventually diagnosed with a mid-shaft tibia-fibula fracture and underwent surgery with nailing. But one month ago, he again met with an accident and was diagnosed with Charcot's foot and equinovarus deformity. He returned to Acharya Vinoba Bhave Rural Hospital (AVBRH), Sawangi, Wardha, Maharashtra, for further management. K-wire was applied for the fixation of Charcot foot with equinovarus deformity. Further on, rehabilitation was started to restore mobility, regain full range of motion and develop muscle strength.

## Introduction

In people with sensory neuropathy, Charcot neuroarthropathy (CN) is a progressive, non-infectious neuro-osteoarthropathy of the bones and joints that destroys the foot's structure [1]. It is an inflammatory disorder that causes osteolysis and is indirectly to blame for the progressive fractures and numerous joint displacements that define its presentation [2]. Although it can also occur in the forefoot and hindfoot, the midfoot is where it most frequently manifests. Congenital neuropathy, infection, toxic exposure, rheumatoid arthritis, multiple sclerosis, tabes dorsalis, traumatic injury, metabolic abnormalities, and syringomyelia are other conditions linked to CN [3]. However, over recent years, diabetes mellitus has emerged as the more typical etiology. Prior estimates of CN's precise prevalence in individuals with diabetes mellitus ranged from 0.1 to 0.4%; more recent estimates range from 0.08 to 0.13% [4]. Up to 7.5% of cases of CN with diabetes and neuropathy-related foot and ankle deformities involve bilateral involvement, and the rate is higher [5]. To select a treatment plan, it is critical to recognize the underlying causes of CN. There is no agreement on the pathologic process that causes CN, and the disorder's origin needs to be better defined [1]. Lack of protective sensation causes the Charcot process to be active and delays the detection of bone injuries that could overload the insensate limb [6]. Sensation loss prevents the affected person from implementing typical protective behaviors, such as activity alteration, off-loading, and seeking medical care [7]. Autonomic dysfunction, neurological, cardiovascular, musculoskeletal, and radiography components are among the clinical findings used to diagnose.

The most frequently acquired lower extremity deformity after trauma to a foot is equines foot deformity (EFD). This condition is characterized by a downward deformity of the ankle, typically accompanied by an internal foot rotation that results in varus-supination. The physiological anatomy of the foot is sometimes further impacted by clawed toes [8]. EFD is the main reason for impairment in people with trauma to the foot because it alters normal gait patterns, which causes pain, impairs ankle stability and passive dorsiflexion during the stance phase, and limits foot clearing during the swing phase [9]. This deformity harms the patient's quality of life. It significantly lowers their chances of returning to a normal life because they require orthotic device assistance with transfers and are at an increased risk of falling [10,11]. One of the initial suggested treatments is physical therapy because it is a significant part of the non-invasive methods used to manage post-traumatic pain [12]. An orthopedic deformity known as post-traumatic talipes equinovarus is brought on by high-energy foot injuries and typically requires correction via surgery [13]. Adduction, supination, varus, and equinus deformities of the foot are symptoms of a condition that occurs after severe trauma to the bone and soft tissues. It has become accepted that the deformity should be treated appropriately as soon as possible to prevent future disabilities, pain, and unease in the foot [14]. For the individual who also has major equinus contracture and concurrent recurrent plantar ulceration, Achilles tendon lengthening should be considered [15]. The proper position of the ankle, the backfoot, the midfoot, and the forefoot can be improved by lengthening the Achilles tendon or reversing the gastrocnemius tendon recession [16].

## Case presentation

A 38-year-old male, farmer by occupation, right-sided dominant, resident of Yavatmal, height and weight of 168 cm and 65 kg, complained of pain in his right foot for 10 days. He gave an alleged history of slipping and falling from a two-wheeler at Asta, Digras, one month ago, sustaining an injury to his right ankle and foot. The pain was sudden, sharp, and progressive. He went to a local hospital, where medication was prescribed. He got relief from the drug. The swelling was sudden in the beginning and subsided over 10 days. But 10 days ago, the patient complained of pain, which is dull, aching, and aggravated by walking, running, or while performing activities of daily living (ADL), and was relieved of medication and rest. The pain is also associated with a tingling sensation in the right lower limb. He had a history of diabetes for five years. He also had a history of mid-shaft tibia-fibula fractures, which he managed with nailing two years ago. With the above complaint, he visited Acharya Vinoba Bhave Rural Hospital (AVBRH), Sawangi, Wardha, Maharashtra. An X-ray was done, and he was diagnosed with a Charcot foot and an equinovarus deformity of the right foot, where he was advised to undergo surgery. Soft tissue reconstruction and tendon-Achilles lengthening with a calcaneocuboid wedge osteotomy of the right side were performed. Post-operatively, the patient was treated with medications. Then, the patient was recommended for physiotherapy for rehabilitation, which aims to regain full mobility, develop muscle strength, and improve quality of life.

Clinical findings

The patient was seen in supine lying and was conscious and well-oriented. Verbal consent was obtained from the patient before the physical examination. On inspection, it was observed that the right hip was slightly abducted and extended, the knee was extended, and the ankle was in equinus and varus deformity. The right leg was bandaged from mid-thigh to toes. Previous surgery marks were seen on the right leg. On palpation, the local temperature was not raised. No bony tenderness was present. Grade 2 tenderness was marked over tendo-Achilles insertion, and severe pain was present at the operative site; the visual analog scale (VAS) score was 8/10 for activity and 3/10 for rest.

Medical management 

**Figure 1:**
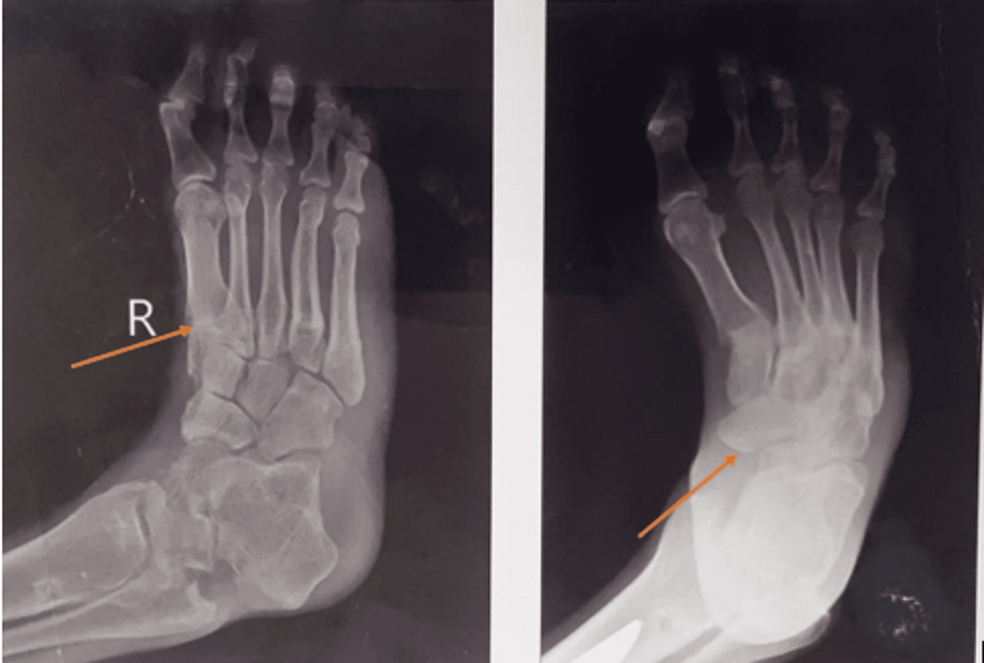
Pre-operative X-ray of Charcot's foot with equinovarus deformity.

**Figure 2:**
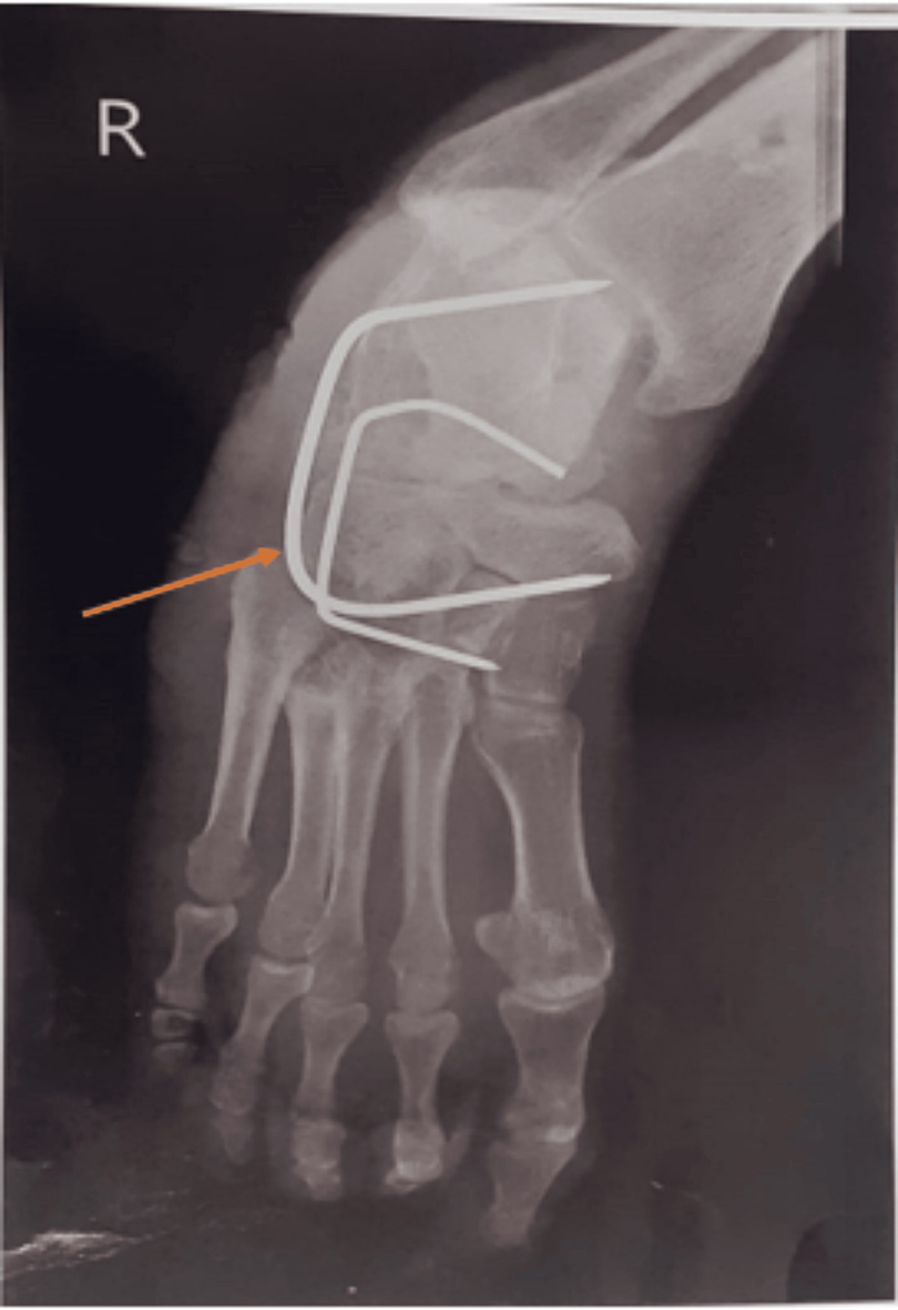
Post-operative X-ray of Charcot's foot with equinovarus deformity with k-wire.

**Figure 3:**
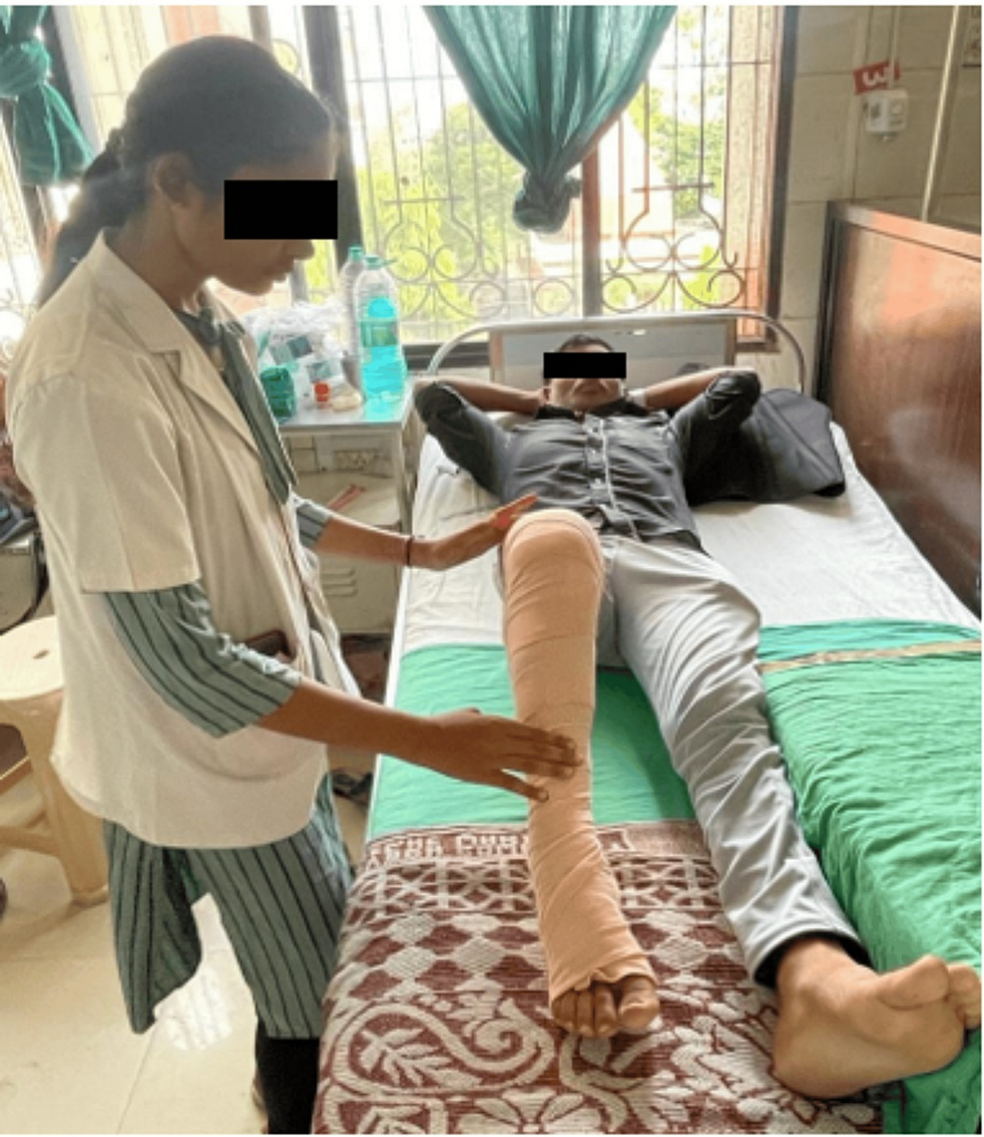
Active-assisted heel slides to improve knee range of motion.

**Figure 4:**
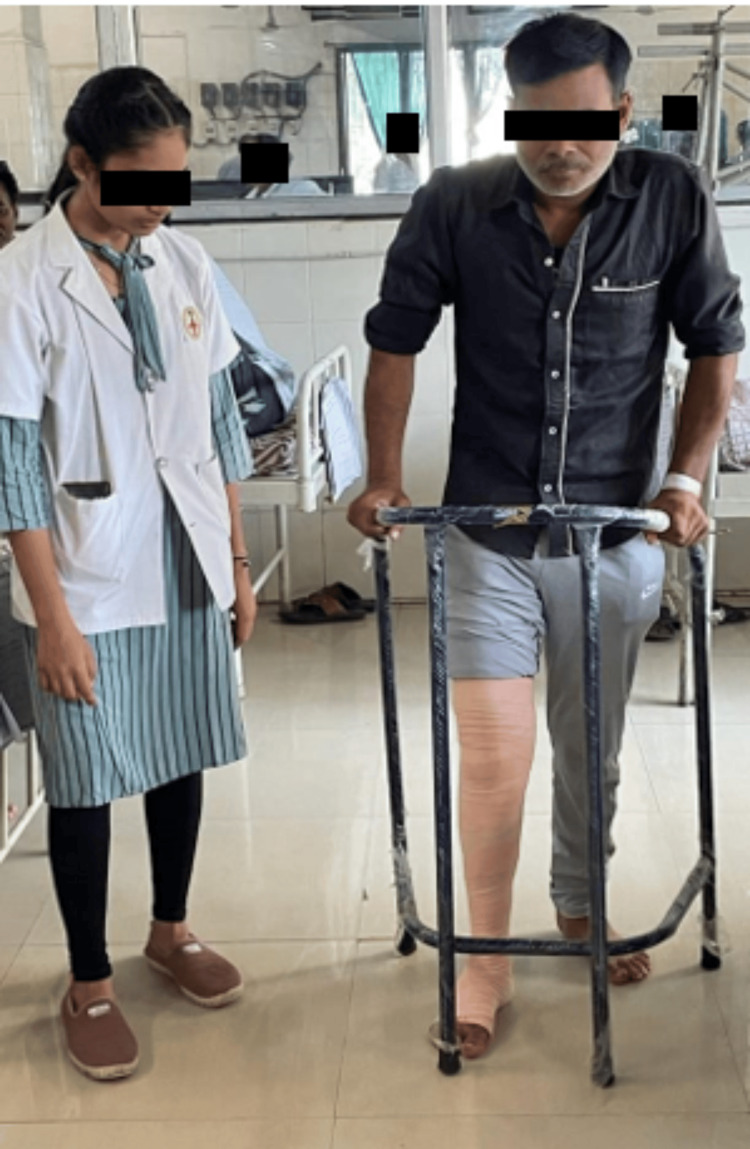
Gait training with walker.

The patient visited AVBRH with major complaints of pain and difficulty walking, due to which he was admitted. An X-ray of the right ankle joint revealed a Charcot foot with an equinovarus deformity. A cast was applied, and after three days, he underwent surgery, soft tissue reconstruction and tendon-Achilles lengthening with a calcaneocuboid wedge osteotomy of the right side. After surgery, postoperative medication is shown in Table 1. A pre-operative X-ray is shown in Figure 1. A post-operative X-ray is shown in Figure 2. The timeline is shown in Table 2. The range of motion at baseline is offered (Table 3). Content of motion post three weeks of rehabilitation led (Table 4). Week-wise goals, physiotherapy management, and dosage are shown in Table 5. The heel slide is shown in Figure 3. Gait training with the walker is shown in Figure 4.

**Table 1:** Post-operative medications. IV: intravenous, SOS: Si opus sit (if needed), AQ: aqua (water), NS: normal saline.

Inj. Ceftriaxone 1 mg IV twice daily
Inj. Neomol 100 ml IV SOS
Inj. Dynapar AQ 1 ml in 100 ml NS IV twice daily
Tab Chymoral Forte thrice daily
Tab Calcium 500 mg twice daily
Tab Limcee 500 mg twice daily

**Table 2:** Timeline of events.

Events	Dates
Date of incidence	17/6/2023
Visited hospital	14/7/2023
Diagnose with Charcot foot and equinovarus deformity	15/7/2023
Underwent surgery	18/8/2023
Referred physiotherapy	19/8/2023
Physiotherapy started	19/8/2023

**Table 3:** Range of motion at baseline.

Joint	Right active	Right passive	Left active	Left passive
Hip				
Flexion	0-80°	0-85°	0-110°	0-120°
Extension	0-10°	0-15°	0-30°	0-30°
Abduction	0-20°	0-25°	0-45°	0-45°
Adduction	0-15°	0-20°	0-30°	0-30°
Knee				
Flexion	0-10°	0-15°	0-125°	0-130°
Ankle				
Plantar flexion	0°	0°	0-40°	0-45°
Dorsiflexion	0°	0°	0-10°	0-15°

**Table 4:** Range of motion post three weeks of rehabilitation.

Joint	Right active	Right passive
Knee		
Flexion	0-120°	0-125°
Ankle		
Plantar flexion	0-35°	0-40°
Dorsiflexion	0-10°	0-15°

Therapeutic management

The patient was referred to the physiotherapy department. The rehabilitation was planned in phases and administered accordingly.

**Table 5:** Weekwise goals, physiotherapy management and its dosage. ROM: range of motion, SLR: straight leg raise, LL: lower limb.

Phases	Goals	Physiotherapy management	Dosage
Phase 1 (0-7thday)	To reduce pain and inflammation, reduce edema, restore mobility, increase range of motion strengthen the lower limb muscles, prevent bedsores.	Ice pack	Thrice a day
		Breathing exercise	10 reps/day
		Ankle pumps	10 reps/day
		Isometrics of quadriceps, hamstring and back	10 reps/day
		Upper limb strengthening	10 reps/day
		Unilateral bridging	10 reps/day
		Left LL ROM exercises	10 reps/day
		Assisted SLR exercise of right LL	10 reps/day
Phase 2 (8th-14th day)	To increase the range of motion, strengthening the lower limb muscles, and stretching of the appropriate group of muscles.	Left LL strengthening (weight cuffs of 2 kg)	10 reps/day
		Dynamic quads (weights)	10 reps/day
		Abdominal crunches	10 reps/day
		Single-leg assisted squats	10 reps/day
		Stretching of hamstring and quadriceps muscle	Five reps with 15 s hold/day
Phase 3 (14-21st day)	Initiation of partial weight-bearing, balance training, gait training and increasing the weight-bearing gradually over the weeks	Russian curls	10 reps/day
		Partial-weight-bearing started using crutches	
		Full weight-bearing with walker then without walker	One round

Outcome measure

The pre- and post-physiotherapy scores of the lower extremity functional scale and visual analog scale are given in Table 6.

**Table 6:** Before and after physiotherapy scores of the lower extremity functional scale and visual analog scale. POD: post-operative day.

Scales	POD-1	POD-21
Lower extremity functional scale	10/80	50/80
Visual analog scale	8/10	2/10

## Discussion

People with peripheral neuropathy are susceptible to the serious complication known as Charcot foot, which is more common in people with diabetes mellitus. It impacts the ankle and foot's soft tissues, joints, and bones [1]. Bones can break as they deteriorate. Immobilization of the affected foot or ankle is the initial and most crucial form of treatment. Charcot foot treatment under conservative management may take a long time [3]. All diabetic patients with Charcot foot treatment should receive routine foot care from a foot and ankle specialist or a diabetic foot problem expert. Surgery is another option for treatment, though it's still debatable whether it's appropriate in the acute or chronic Charcot phase [4]. During the acute phase, a barrage of inflammatory mediators and cytokines encourages edema and bone resorption and fragmentation. Surgery off-loading is the main justification for Charcot reconstruction, which aims to stop the progression of deformities and ulcers.

A complex congenital foot deformity called post-traumatic congenital talipes equinovarus (CTEV), or clubfoot, can restrict a person's mobility because it makes walking challenging. Clinical evidence includes a resting foot deformity called equinovarus [16]. The Ponseti method of casting is typically used in treatment. Additional therapies could involve an Achilles tenotomy, bracing, or surgery. Immobilization, consistent foot care, and surgery are typical physiotherapy remedies for Charcot's foot [7]. Surgical and physiotherapy interventions are important in treating post-traumatic congenital talipes equinovarus. A systematic review suggests that exercise might help people with Charcot-Marie-Tooth disease (CMT) maintain their strength and function. Enhancement in strength, functional activities, and physiological adaptations after exercise were among the notable effects that were reported [17]. There were not many studies available, and the evidence was of mediocre quality. Uncertainty surrounds the best type and level of exercise for individuals with CMT as well as the long-term effects of exercise [18]. Exercises that extend the foot are advised to avoid Achilles tendon shortening [19]. The primary objectives of physiotherapy included strengthening the quadriceps and hamstrings, patient education, secondary complication prevention, and maintenance. The patient's pain has subsided, and they can now walk unassisted. The patient has returned to their usual walking pattern. Individualized treatment plans must be developed based on a thorough examination, a detailed history, and clearly stated patient objectives [17]. Physical therapy management is a crucial part of rehabilitation to resume daily life and enhance the quality of life.

## Conclusions

Physical therapy is essential to the all-encompassing care for Charcot neuroarthropathy and post-traumatic CTEV. Although the emphasis may differ depending on the treatment condition, both include early intervention, individualized treatment plans, exercises, orthotic devices, education, and cooperation with other specialists. The intention is to help individuals with these conditions function better, avoid complications, and improve their general health. According to the case study above, a traditional surgical procedure coupled with timely planned physical therapy rehabilitation helped patients gradually improve their functional goals, a crucial component of full recovery in these post-operative cases. Planning before surgery must be comprehensive and in-depth. Stable fixation and continued articular congruity make early mobilization and a stronger practical performance possible.
